# CovidShiny: An Integrated Web Tool for SARS-CoV-2 Mutation Profiling and Molecular Diagnosis Assay Evaluation In Silico

**DOI:** 10.3390/v15102017

**Published:** 2023-09-28

**Authors:** Shaoqian Ma, Gezhi Xiao, Xusheng Deng, Mengsha Tong, Jialiang Huang, Qingge Li, Yongyou Zhang

**Affiliations:** 1 The State Key Laboratory of Cellular Stress Biology, National Institute for Data Science in Health and Medicine Engineering, Research Center of Molecular Diagnostics of the Ministry of Education, School of Life Sciences, Xiamen University, Xiamen 361100, China; msq21@mails.tsinghua.edu.cn (S.M.); ggwp233@hotmail.com (G.X.); dengxsh@stu.xmu.edu.cn (X.D.); mstong@xmu.edu.cn (M.T.); jhuang@xmu.edu.cn (J.H.); qgli@xmu.edu.cn (Q.L.); 2 Faculty of Medicine and Life Sciences, Xiamen University, Xiamen 361100, China

**Keywords:** SARS-CoV-2, mutation analysis, integrated web tool, assay evaluation

## Abstract

The coronavirus disease 2019 (COVID-19) pandemic is still ongoing, with severe acute respiratory syndrome coronavirus 2 (SARS-CoV-2) continuing to evolve and accumulate mutations. While various bioinformatics tools have been developed for SARS-CoV-2, a well-curated mutation-tracking database integrated with in silico evaluation for molecular diagnostic assays is currently unavailable. To address this, we introduce CovidShiny, a web tool that integrates mutation profiling, in silico evaluation, and data download capabilities for genomic sequence-based SARS-CoV-2 assays and data download. It offers a feasible framework for surveilling the mutation of SARS-CoV-2 and evaluating the coverage of the molecular diagnostic assay for SARS-CoV-2. With CovidShiny, we examined the dynamic mutation pattern of SARS-CoV-2 and evaluated the coverage of commonly used assays on a large scale. Based on our in silico analysis, we stress the importance of using multiple target molecular diagnostic assays for SARS-CoV-2 to avoid potential false-negative results caused by viral mutations. Overall, CovidShiny is a valuable tool for SARS-CoV-2 mutation surveillance and in silico assay design and evaluation.

## 1. Introduction

The COVID-19 pandemic continues to spread worldwide, with over 767 million confirmed cases as of 12 July 2023 [1]. Recent studies have shown that new SARS-CoV-2 variants, such as BF.7, BQ.1, BQ.1.1, XBB.1, XBB.1.5, BA.2.75, EG.5, and BA.2.86, may increase transmissibility and evade immunity, potentially reducing the efficacy of current vaccines such as BNT162b2 and neutralizing antibody-based therapies [2,3,4,5,6]. These changes may be caused by new mutations introduced to critical protein domains such as RBD in spike protein. For instance, the K444T and N460K mutations found in BQ.1, along with the additional R346T mutation in BQ.1.1, could potentially enhance the antibody and serum resistance of the variants by interfering with antibody recognition without affecting the affinity to ACE2 [7,8,9,10]. V445P mutation observed in XBB and XBB.1 variants might induce similar effects on antibody resistance, but the introduction of F486S and R493Q mutations slightly reduces the ACE2 affinity [8,10]. Other variants, such as BA.2.75, BF.7, and XBB.1.5, exhibit distinctive combinations of mutations that contribute to their protein stability, receptor affinity, and immune evasion [9,11,12]. Recent variants such as EG.5 and BA.2.86 contain F456L or P681R mutations that may cause immune evasion from XBB.1.5 neutralizing antibody or enhance the ability to penetrate cell membrane [13,14,15,16]. With the spreading and evolving of variants, more mutations will be introduced and new variants will have different characteristics, challenging the assay and vaccine development for SARS-CoV-2.

Therefore, access to the real-time mutation profile of SARS-CoV-2 is essential for scientists and public health officials to study variant characteristics, evaluate high-risk variants, and implement better assays. Several tools are available to track the number of infected cases of SARS-CoV-2 in real-time, perform mutation and phylogenetic analyses of the virus, or track transmission [17,18,19,20]. Some platforms use computational methods to predict the pathogenicity of specific mutations [21,22,23]. Given the crucial role that the three-dimensional structure of RNA plays in the viral genome, along with its specific secondary and tertiary interactions, significant efforts are being invested by the RNA-Puzzles community in developing prediction pipelines for the 3D structure of the SARS-CoV-2 genome [24,25]. For instance, Gumna et al. developed a computational pipeline that is utilized for the reference-free analysis of the 3′UTR and 5′UTR 3D structures of the SARS-CoV-2 genome [24]. This pipeline has the potential to facilitate comparative analyses of viral RNA homologs, thereby enhancing the reliability of function predictions [24]. The interactive web tool developed by Dong et al. [17] is a powerful tool for assessing the current transmission situation of SARS-CoV-2. However, most tools are command-line-based and can be challenging for users without professional bioinformatics assistance.

Moreover, a comprehensive and user-friendly tool that integrates mutation annotation and applications in a clinical setting—for example, the design and evaluation of diagnostic assays such as real-time quantitative reverse transcription polymerase chain reaction (qRT-PCR) assays in silico—is still lacking. Such a mutation profile tool for viral genome can be used in the auxiliary design of qRT-PCR-based molecular assays [26]. The qRT-PCR assay is considered the gold standard for virus detection, due to its high sensitivity and specificity [27,28]. Most qRT-PCR assays for SARS-CoV-2 have been developed using primer/probe sets recommended by the World Health Organization (WHO). These assays target four commonly selected regions: ORF1ab, E, N, and S genes. Among these, the N gene stands out as the most widely used genomic target region for assay development [28,29,30]. By 6 March 2023, the United States Food and Drug Administration (FDA) had granted emergency utilization authorization (EUA) to over 456 molecular diagnostic tests [31]. However, the performance of these assays varies greatly, with the lowest detection limit (LoD) ranging from 180 nucleic acid amplification tests detectable units (NDU)/mL to 600,000 NDU/mL [32]. Numerous factors, including virus mutations, can impact the sensitivity of a molecular diagnostic assay [33,34]. The decreasing in sensitivity due to the virus’s mutation even yields a severe false-negative result [35]. For instance, the B.1.1.7 lineage (501Y.V1 variant), containing the 69-70del mutation, can cause a “dropout” effect on S gene assays when tested with the TaqPath COVID-19 assay [36,37]. Thus, an in silico evaluation tool for assessing assay coverage of SARS-CoV-2 across the country/region is urgently required [38]. 

**Figure 1:**
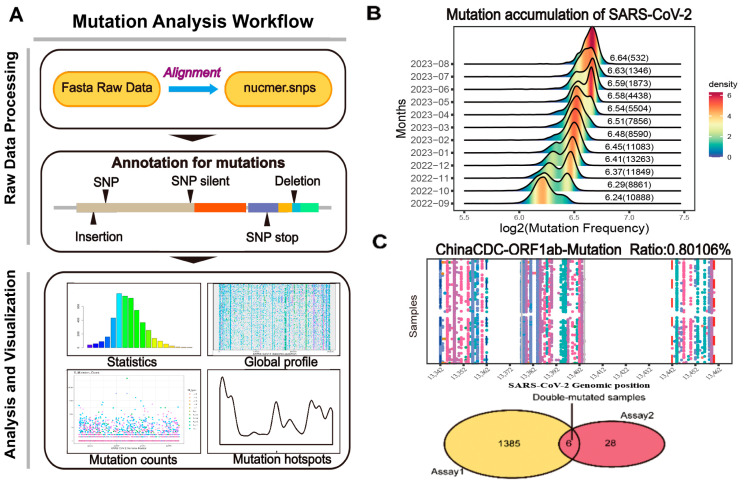
**The framework of CovidShiny.** (**A**) Mutation analysis workflow of CovidShiny. After alignment with the query sequence with the reference sequence, the output contains the position of all mutations of SARS-CoV-2. Mutation annotation includes identifying mutational events (SNP, insertion, deletion, etc.). (**B**) The density ridgeline plot of mutation-frequency accumulation since September 2022. The number shown beside the ridge is the log2 transformed average mutation counts of SARS-CoV-2 in a month (with a total sample number per month in brackets). (**C**) RT-PCR assay validation. With primers binding sites and the total number of virus samples available, assays are evaluated according to their potential in detecting coverage in large-sized SARS-CoV-2 samples, since assays targeting regions with continuing-mutating patterns may lead to confusing results. Samples carrying double mutations in double-assay are also presented in CovidShiny.

Since 27 January 2020, we have continuously monitored the mutation of SARS-CoV-2. In this regard, we introduce CovidShiny, an all-encompassing tool that profiles SARS-CoV-2 mutations, evaluates diagnostic assays based on nucleotide sequences in silico, and provides a hub for downloading mutation-related data (Figure 1A and Figure S1). With the CovidShiny application, users can conveniently track the mutation dynamics of SARS-CoV-2 and forecast the spatiotemporal expansion of emerging mutation lineages. Additionally, the tool’s functionalities enable users to visualize global or regional mutation profiles in the genomic region targeted by sequence-based assays and facilitate the evaluation and improvement of molecular diagnostic assay design (Figure 1C). We deployed an instance so that users could access it at http://www.zhanglabtools.online/shiny/CovidShiny/. Users could also deploy CovidShiny locally by downloading the source code and database from our github.

## 2. Materials and Methods

### 2.1. Data Collection, Pre-Process, and Curation

We retrieved the most current genome sequences of SARS-CoV-2 from GISAID (26 June 2023) [39] for our web-based application. A total of 245,0328 (after filtered in UNIX) high-coverage and complete (>29,000 nucleotides) genomes of SARS-CoV-2 were retrieved and aligned with the first-published SARS-CoV-2 genome (MN908947.3) and the alignment accuracy was checked manually base-by-base. We will maintain the application and update the data monthly during the COVID-19 pandemic.

The CovidShiny App has been developed using the R programming environment. It is based on the *Shiny* framework (https://cran.r-project.org/web/packages/shiny/ (accessed on 22 November 2022)) running on R version 4.3.0. The full dataset of mutations listed in CovidShiny is derived from genomic sequences downloaded from GISAID (https://www.gisaid.org/ (accessed on 22 November 2022)), followed by data cleaning and alignment using *seqkit* software (*v2.5.1*) [40] and *NUCmer* (NUCleotide MUMmer, v4.0.0rc1) [41], respectively, under UNIX (the analysis script is available on Github: https://github.com/catsingchannel/CovidShiny (accessed on 22 November 2022)). 

The result of single nucleotide polymorphisms (SNPs) (.snps file) was further processed by removing special characters and duplicates in the *snps-porocess* C++ program. We divided the app into several *Shiny* modules. These modules were organized together by the server function in the *app.R* script. Meanwhile, the visualization and function design of the modules were implemented separately. The mutation events, such as the insertion and deletion of multiple nucleotides in separate records, were merged into a single record and then annotated using the GFF3 file as the reference sequence in R. The *reactive* function of *Shiny* interactively processes users’ submitted assay sequences. All the input parameters are automatically processed by the *eventReactive* function activated by the submit button. The whole process ensures the privacy of submitted sequence information. The dataset filtered by the user’s setting (by location, time, variant, and other parameters) in the data table interface is also available for download.

### 2.2. Interactive Modules

#### 2.2.1. Mutation Statistics

This module provides an overview of SARS-CoV-2 mutation features and displays mutation statistics in terms of mutation ridgeline density plots for each country, a summary of mutational events, and mutation counts for every single gene. The ridgeline-density plot provides a quick and global mutation profile for the user-defined genomic region. Time-course tracking of geographical mutation distribution is also presented.

#### 2.2.2. SNP and Protein Profile

This module displays the SNPs and proteins mutation profile in the SARS-CoV-2 genome as a heatmap or counts figure. Users can adjust the geographic location parameter to compare mutation patterns in different countries or regions. A dynamic table is generated in the SNP/protein profile panel corresponding to the figure above for users to download and explore. Users can also select variants of interest in specified regions and submission dates to analyze co-mutations in the ‘*UpsetR plot*’ section.

#### 2.2.3. Assay Profile

This module implements the evaluation and comparison of currently approved or user-defined sequence-dependent diagnostic assays, particularly RT-PCR assays. Within each panel, we offer a display demo, allowing users to input relevant sequences or standardized assay lists to determine the mutation ratio in various geographic locations. The mutation ratio is calculated based on the number of samples with mutations in the primer/probe binding site within a specified country location (or globally), relative to the total number of samples recorded in that location (or globally). Additionally, users can download tables containing mutation information. The assays-comparison submodule requires a user-provided list of RT-PCR assay information in .txt format (see the web homepage). Users can set parameters, such as geographic location, for comparison.

#### 2.2.4. Last Five Nucleotides of Primers

This module implements the calculation of the mutation ratio in SARS-CoV-2 genomic binding sites for the last five nucleotides of RT-PCR primers, which are supposed to be the most sensitive to mutations for a successful polymerase chain reaction (PCR).

#### 2.2.5. Double Assay Evaluation

Most approved SARS-CoV-2 molecular diagnosis assays detect more than one genomic region to avoid the potential false-negative result. Therefore, we developed the *double-assay* submodule, which allows users to check the mutation and coverage of the most commonly used double assays or the user-defined double assays. Samples carrying mutations in the primer/probe region are recorded in the table below the figure, which can be downloaded for further analysis.

#### 2.2.6. RT-PCR Primer Design

This module uses *msa* and *rprimer* R packages to automatically generate the RT-PCR assay for chosen variants and target genes [42,43]. Mutation profiles of selected variants filtered by region and sample submission date are used to construct genome sequences for the corresponding variant. Mutations with a detection rate in samples exceeding one thousandth are incorporated into the genome sequence construction, with the most prevalent mutation at a specific position being prioritized. Genome sequences for each variant are aligned by default settings of the *msa* package and, then, used as input for the *rprimer* package. The generated assays are further filtered by Tm and GC content difference between forward and reverse primers and the best assay is selected for display.

#### 2.2.7. Structure Viewer for Mutated Proteins

To assist the development and in silico evaluation of antigen-antibody-based SARS-CoV-2 diagnosis assay, we developed the *Strucview* module to label and visualize mutated position in the N or S proteins of customized or recently collected samples, based on homology modeling. This module also provided visualization of the homology models obtained through SWISS-MODEL [44,45] and mutated position labels for the N and S proteins of common SASR-CoV-2 variants. The template used for homology models is obtained from PDB (PDB ID: 7JWY) for S protein and predicted by I-TASSER [46] for N protein.

## 3. Results

**Figure 2:**
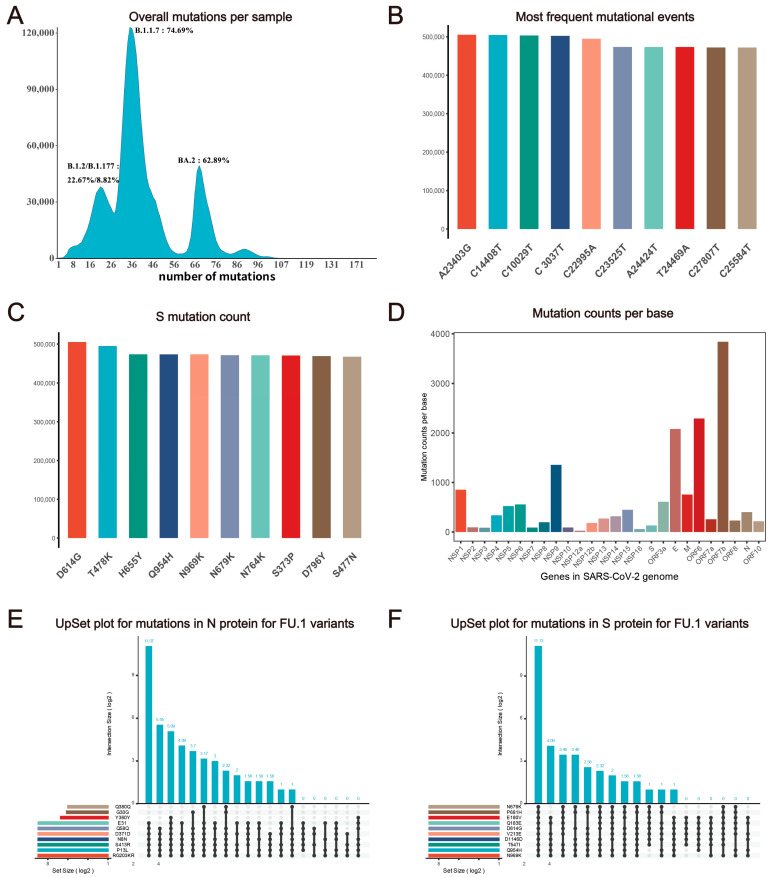
**Statistics description of SARS-CoV-2 mutation features.** (**A**) Distribution of mutation counts per sample. The x-axis represents the number of mutations and the y-axis represents the number of samples with a corresponding mutation number. Variants with the largest sample number in peaks and their percentage are labeled around each peak. (**B**) Frequency of top 10 nucleotide variant types globally. (**C**) The frequency of mutational events shows the top 10 popular variants in SARS-CoV-2 spike protein. (**D**) Average mutation counts per base in genes. (**E**) UpsetR plot of mutations (N protein of all FU.1 variant samples; only the top 10 mutations are shown). (**F**) UpsetR plot of mutations (S protein of all FU.1 variant samples; only the top 10 mutations are shown).

Despite having a lower mutation rate than most RNA viruses, SARS-CoV-2 accumulates mutations resulting in genomic diversity within and between infected patients, necessitating continuous monitoring. Analysis indicates an increase in the mutation ratio from a log2 transformed average mutations per sample of 6.24 in September 2022 to 6.84 in August 2023 (Figure 1B and Figure S2), with an overall mutation count of approximately 41.09 per sample until 26 June 2023 (Figure 2A). We computed statistics on SARS-CoV-2 mutation features and identified the top 10 common SNP types across all samples (Figure 2B). Furthermore, a global analysis of protein mutation types is essential for drug and vaccine development, as mutations in the spike protein can impact vaccines like BNT162b2 and neutralize antibody-based therapy, such as bamlanivimab and imdevimab (Figure 2C) [47,48]. Additionally, some genomic regions of SARS-CoV-2, such as ORF7b, ORF6, and E gene, are at a higher risk of mutation, indicating the continual evolution of mutation hotspots in these areas (Figure 2D).

We developed the ‘*UpsetR plot*’ submodule on our website to investigate the potential overlap between different variants of SARS-CoV-2. We observed that five N protein mutations (S413R, Q58Q, RG203KR, P13L, D371D, N8N) are highly correlated in some variants, based on the protein mutation profile from our web tool (e.g., FU.1, see Figure 2E). Since the N protein is commonly used as the antigen test target, antibody targeting sites should avoid co-mutation signatures. Users can also select pangolin lineage or GISAID clade of interest on CovidShiny to investigate potential co-mutations. Co-occurring mutations in the same sample may have a potential correlation for impacts on genomic RNA folding, mRNA structure, and changes in biological functions, such as transcription and translation efficiency, as well as in replication rates [49]. Combined the mutation profiles and analysis results such as those of the UpsetR plot can be helpful for guidance of assay validation and new assay design (Figure 2E,F) [26].

**Figure 3:**
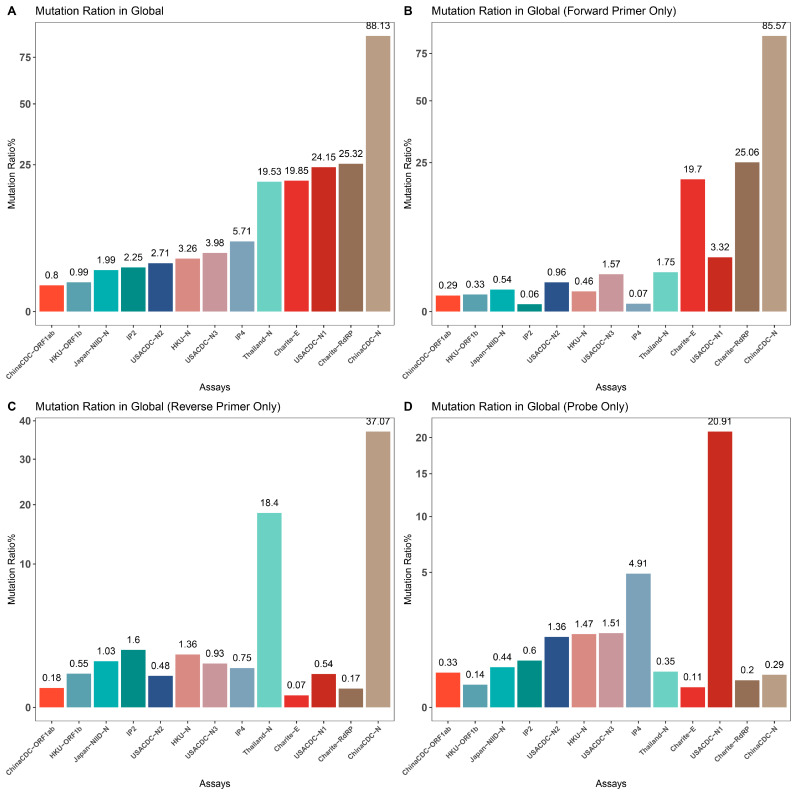
**Mutation ratio for the demo assays.** We calculated the mutation rate in primers and probe binding regions for popular assays and summarized them for comparison. (**A**) Overall mutation rate for primer and probe binding regions for each assay. (**B**) Mutation rate for forward primer binding region for each assay. (**C**) Mutation rate for reverse primer binding region for each assay. (**D**) Mutation rate for probe binding region for each assay.

**Figure 4:**
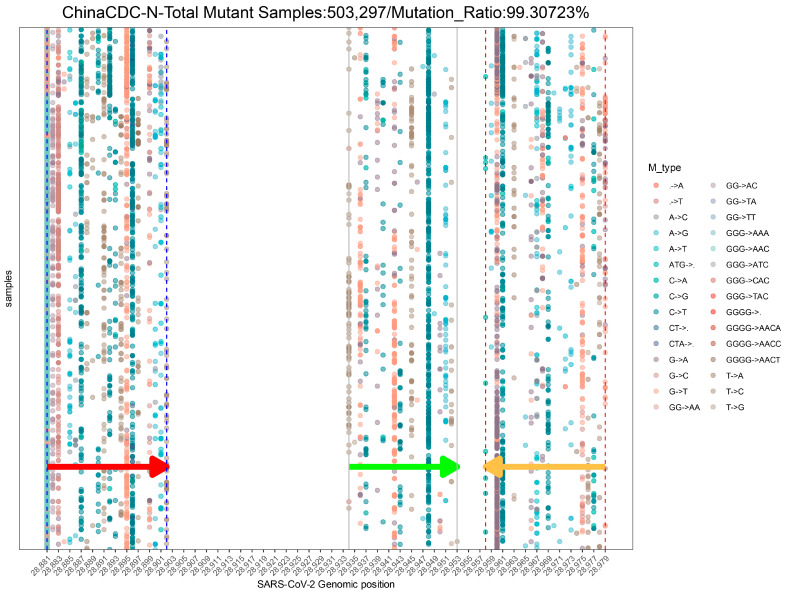
**The demo mutation profile in the genomic binding sites of ChinaCDC-N primers and probe globally.** This profile uses samples collected from 1 January 2022 to 26 June 2023. Only the top 30 mutation types with the highest occurrence frequency are shown in this profile. The arrows indicate the location and direction of primers or probes (red arrow: forward primer; green arrow: probe; orange arrow: reverse primer).

**Figure 5:**
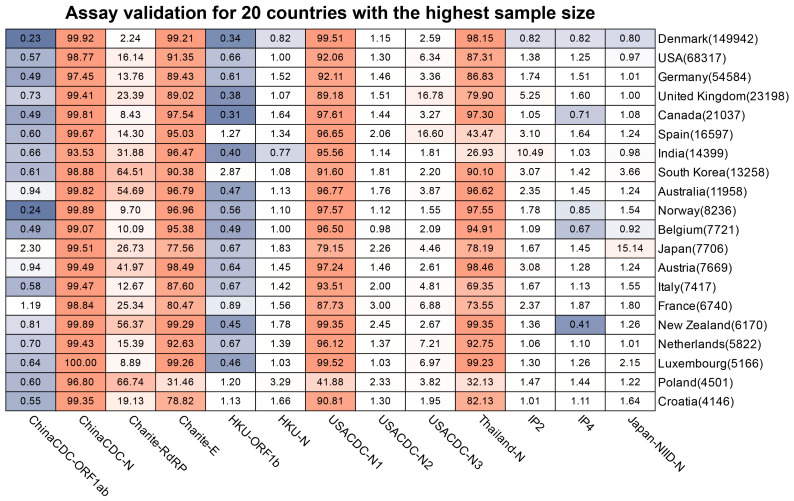
**Mutation ratio of commonly used assays across the countries and regions.** We selected countries and regions with the highest sample size that was submitted after 1 January 2022, to draw the heatmap. The red color indicates the severity of mutations in the assay (for the related country). The blue color indicates the less severity of mutations in the assay (for the related country). The number in the bracket next to the country is the total of the samples available.

To assess the impact of SARS-CoV-2 mutations on molecular diagnosis, we analyzed the genomic region targeted by commonly used diagnostic assays (Supplementary Table S1) [27,50,51,52,53,54,55]. The mutation ratio for these assays varied widely, ranging from 0.8% (ChinaCDC-ORF1ab) to 88.27% (ChinaCDC-N) (Figure 3). We observed an enriched mutation pattern (28881: GGG → AAC) in the 5′-end of the ChinaCDC-N forward primer, which accounted for most of the mutations in this assay and could potentially affect the assay’s sensitivity (Figure 4). To validate this, we analyzed the mutation rate in the primer binding site using *ComplexHeatmap* and *circlize* R packages [56,57] for all assays on samples submitted after 1 January 2022. Our analysis revealed that the 28,881: GGG → AAC mutation was widespread from Denmark to Croatia (Figure 5), indicating that the ChinaCDC-N assay may not be suitable for COVID-19 testing. Moreover, we observed high mutation ratios in the Charite-RdRP assay targeted region for samples from Poland (66.74%) and Austria (41.97%), and in the Japan-NIID-N assay targeted region for samples from Japan (15.14%) (Figure 5). Our findings emphasize the need to check the geographical mutation status, even for approved diagnostic assays, to provide reliable testing results in the face of ongoing SARS-CoV-2 mutations during transmission.

**Figure 6:**
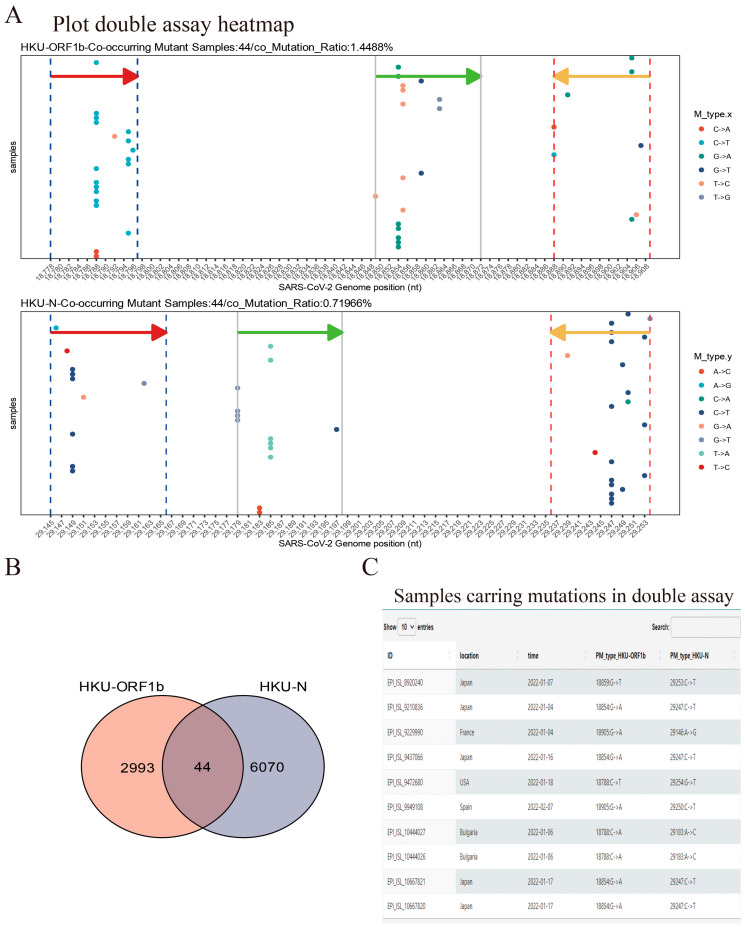
**Demo for *Double-assay* module usage.** (**A**) Samples submitted after 1 January 2022 show mutations occurring in both HKU-ORF1b and HKU-N primers/probe binding sites. The arrows indicate the location and direction of primers or probes (red arrow: forward primer; green arrow: probe; orange arrow: reverse primer). (**B**) Sample counts that contained a mutation in the target site for both assays; 44 samples shown in (**A**) have mutations in both assays—target sites in this case. (**C**) The information table of double-mutated samples is available for download (10 queries are shown).

To assess the coverage of double assays for SARS-CoV-2 samples, we created the *Double-Assay* module. We analyzed samples with mutations in the binding sites of HKU-ORF1b and HKU-N primers/probes (Figure 6 and Supplementary Table S2). The results showed a much lower percentage of samples with double mutations than single assays (Figure 6 and Supplementary Table S3). This highlights the importance of using multiple target assays for any clinical diagnostic to minimize the risk of false-negative results.

**Figure 7:**
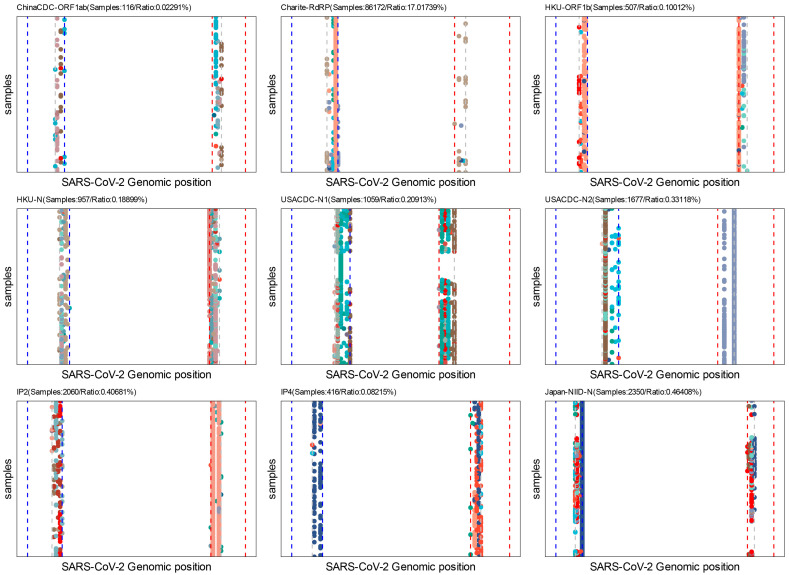
**Batch assay analysis for the last five nucleotides of primers.** Each point in the figure represents a single mutation in the binding sites of the last five nucleotides of forward or reverse primers. Only samples submitted after 1 January 2022 are used in these analyses. Colors indicate different variation types (A- > G, G- > C, etc.). The blue dash line indicates the forward primer and the red dash line indicates the reverse primer; the grey dash line indicates the start of the last five nucleotides for primers.

The last-five-nucleotide binding sites of the primers are expected to be much more important for a successful PCR [58]. To characterize it, we developed the *Last-five Nr-profile* module. Our analysis revealed that the forward and reverse primer binding sites are extensively mutated, particularly in the N gene, which may pose challenges in designing primers for the N gene (Figure 7). Considering that the N gene is still a commonly selected target region for the SARS-CoV-2 qRT-PCR assay, we suggest that the test targeting the N gene still needs more experimental validation [59]. 

Utilizing our mutation profile database, we offer an automatic RT-PCR assay design through the “*rprimer*” package in our web tool. Users can choose a target gene and select up to 50 variants of interest for assay design. For each variant, we construct and align the target gene sequence to generate an optimal assay, which is then displayed. Furthermore, this tool includes supplementary options for users, such as specifying the sample region and submission date, to enhance the optimization of assay design.

**Figure 8:**
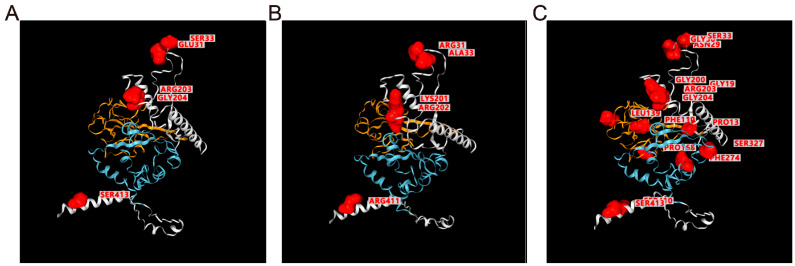
**Structures and mutation labels for SARS-CoV-2 S protein.** (**A**) Nucleocapsid protein structures of original variants (NC_045512.2) predicted by I-TASSER, mutation position for BA.2.75 variants are labeled and highlighted in this view for comparison with (**B**). (**B**) Nucleocapsid protein structures of BA.2.75 variants based on homology modeling using SWISS-MODEL. The mutation position is labeled in red in this view. (**C**) Mutation labels for BF.7 variants based on structures of original variants; note that this view only labeled positions for mutations without any difference in structure. For all structure views, the blue region is the C terminal domain (CTD), and the orange region is the N terminal domain (NTD). The mutated position is marked red.

Antigen-based diagnostic tests are another important type of SARS-CoV-2 assay. The basis of antibody–antigen interaction of such kinds of assays makes false-negative results possible when a mutation occurs in the protein-coding region of the virus genome. To monitor mutations in SARS-CoV-2 N and S proteins, we created the *Strucview* model. The Omicron BA.2.75 variant reveals that mutations are primarily located on regions that are neither the C terminal domain (CTD) nor the N terminal domain (NTD). This finding is consistent with the top mutations in all BF.7 variant samples (Figure 8). These results suggest that the sensitivity of antigen-based tests targeting these regions of the SARS-CoV-2 N protein requires further experimental validation. As such, diagnosis assay developers should not consider this region as a target for antibodies.

## 4. Discussions

CovidShiny is a comprehensive tool for mutation profiling and in silico assay evaluation for SARS-CoV-2. With monthly data updates, users can use CovidShiny to gain more insight into SARS-CoV-2 mutations, with significant medical and biological implications for prevention, diagnosis, and therapy [60]. By utilizing real-time mutation profiles, CovidShiny offers user-friendly tools for RT-PCR-assay evaluation and automatic assay generation, which simplifies the design and assessment of diagnostic assays, vaccines, and therapeutic drugs for SARS-CoV-2. Additionally, CovidShiny can be modified to process and display other the mutation profiles of other viruses. This versatility can remove obstacles for researchers, diagnosis-assay developers, and public health officials seeking complete mutation data and in silico assessment for molecular diagnosis and therapeutic-targeted proteins. 

Despite the benefits of computational/web tools like CovidShiny, their conclusions still require experimental verification, especially for results of assay evaluation and RT-PCR-assay generation. However, the lack of such experimental results for CovidShiny, especially for assay evaluation and the assay-generation module, means the mutations in primer or probe binding site recorded in the mutation profile cannot predict the extent of the loss of assay performance in vitro, and the sensitivity and specificity of generated assays may not meet the in silico expectation.

Conducting PCR amplification in synthetic or actual SARS-CoV-2 samples represents a vital and effective approach for validating computational or statistical predictions. To provide evidence of the analysis and assay-evaluation result, although no experimental results have been shown in our research, we are actively seeking collaboration with other laboratories that are capable of performing RT-PCR experiments based on our analyses or providing real samples for further validation. Such collaboration is expected to substantiate the in silico evaluation of CovidShiny, particularly regarding the automatic-assay-design module. Moreover, both sensitivity and specificity serve as crucial indicators for assay evaluation and selection. Whether performing in vitro assay verification or in silico simulation, it is imperative to consider both of these metrics. However, due to the limited availability of sample data sources, most sequences within the CovidShiny analysis pipeline consist of virus genome sequences. Consequently, the pipeline can only provide information on true-positive or false-negative results. Enhancing the CovidShiny analysis pipeline and database is essential, and one potential method involves incorporating an assay-evaluation module for other virus genomes that are phylogenetically related to SARS-CoV-2 or that exhibit similarities in nucleotide sequences. This expanded evaluation may yield valuable insights into false-positive rates and assay specificity, thereby guiding assay design effectively. Alternatively, the incorporation of synthetic negative samples presents another viable option to implement this functionality.

The update frequency poses a significant challenge for database-based web dashboards, particularly for applications such as CovidShiny, which offers mutation profiles for the SARS-CoV-2 virus, which continues to spread and evolve, with new variants emerging worldwide. While our CovidShiny instance is currently limited to monthly updates, enhancing it to achieve weekly or daily updates could greatly enhance its effectiveness in pandemic monitoring. This is especially crucial for tracking the mutation profiles of newly emerging high-risk variants, such as EG.5 and BA.2.86, as mentioned in the Introduction, which differ significantly from ancestral variants. CovidShiny, built on the Shiny framework, provides portable applications, allowing users to access the source code and deploy it locally with their own sequence data. This feature can partially mitigate the challenges associated with the update frequency of a single website. Therefore, we encourage users to deploy the local server to run the Covidshiny to obtain better user experience. 

Going forward, mutation-profiling applications for SARS-CoV-2 may include enhanced data visualization, improved user experience, and additional features. Enhancing the robustness and result readability of web tools such as CovidShiny is also essential. Through our ongoing maintenance and updates for CovidShiny, we plan to address any defects and deficiencies in the application. Additionally, our updates will focus on improving the readability of output results and the user-friendliness of our functions. This will involve making corrections to annotations and manuals within our application’s interface. 

Moreover, with the emergence of new viruses such as monkeypox, there is a need for more flexible mutation-profiling tools that can adapt to different pathogens. Thus, continuous maintenance and improvement of CovidShiny’s analysis pipeline would enable it to offer mutation profiles for a broader range of viruses. To conclude, CovidShiny can potentially be a valuable tool for monitoring the spread of not only SARS-CoV-2 but also of other infectious diseases.

## Data Availability

We deployed the CovidShiny web app based on the Shiny server, which could be accessed at http://www.zhanglabtools.online/shiny/CovidShiny/. This website will be maintained continuously and mutation data will be updated monthly. The code of the app and analysis script is available on Github: https://github.com/catsingchannel/CovidShiny.

## Supplementary Materials

The following supporting information can be downloaded at: https://www.mdpi.com/article/10.3390/v15102017/s1, Table S1: Primer and probe information for popular commonly used assays for SARS-CoV-2 [27,50,51,52,54,55,61]. Table S2: Samples with mutations occurring in both HKU-ORF1b and HKU-N primers/probe binding sites. Table S3: Mutation ratio for commonly used assays and double mutation ratio for dual assays. Figure S1: Overview and brief annotation for all modules and functions of CovidShiny. Figure S2: The density ridgeline plot of mutation frequency accumulation since 2020-01.
