# Supplementary material for: CovidShiny: An Integrated Web Tool for SARS-CoV-2 Mutation Profiling and Molecular Diagnosis Assay Evaluation In Silico
Source: Viruses. 2023 Sep 28;15(10):2017. doi: 10.3390/v15102017 (PMC10611021; doi:10.3390/v15102017)
Supplement: Supplementary file 1 [file viruses-15-02017-s001.zip › viruses-2600446-supplementary.pdf]

Supplementary Table S1. Primer and probe information for popular Commonly used Assays for SARS-Cov-2.

Institute/Count	Assay	Forward.primer(5' -	Reverser.primer(5' -	Probe(5' - 3')	Referen
China CDC	ChinaCDC-ORF1ab	CCCTGTGGGTTTTACACTTAA	ACGATTGTGCATCAGCTGA	FAM-CCGTCTGCGGTATGTGGAAAGGTTATGG-BHQ1	55
China CDC	ChinaCDC-N	GGGGAACTTCTCCTGCTAGAAT	CAGACATTTTGCTCTCAAGCTG	FAM-TTGCTGCTGCTTGACAGATT-BHQ1	
Charite	Charite-RdRP	GTGARATGGTCATGTGTGGCGG	CARATGTTAAASACACTATTAGCATA	FAM-CCAGGTGGWACRTCATCMGGTGATGC-BHQ1	27
Charite	Charite-E	ACAGGTACGTTAATAGTTAATAGCGT	ATATTGCAGCAGTACGCACACA	FAM-ACACTAGCCATCCTTACTGCGCTTCG-BHQ1	
HKU	HKU-ORF1b	TGGGGYTTTACRGGTAACCT	AACRCGCTTAACAAAGCACTC	FAM/ZEN-TAGTTGTGATGCWATCATGACTAG-IBFQ	54
HKU	HKU-N	TAATCAGACAAGGAACTGATTA	CGAAGGTGTGACTTCCATG	FAM/ZEN-GCAAATTGTGCAATTTGCGG-IBFQ	
US CDC	USACDC-N1	GACCCCAAAATCAGCGAAAT	TCTGGTTACTGCCAGTTGAATCTG	FAM-ACCCCGCATTACGTTTGGTGGACC-BHQ1	61
US CDC	USACDC-N2	TTACAAACATTGGCCGCAAA	GCGCGACATTCCGAAGAA	FAM-ACAATTTGCCCCCAGCGTTAG-BHQ1	
US CDC	USACDC-N3	GGGAGCCTTGAATACACCAAAA	TGTAGCACGATTGCAGCATTG	FAM-AYCACATTGGCACCCGCA ATCCTG-BHQ1	
Thailand	Thailand-N	CGTTTGGTGGACCCTCAGAT	AATGGAGAACGCAGTGGGG	FAM-CAACTGGCAGTAACCA-BQH1	62
Paris	IP2	ATGAGCTTAGTCCTGTTG	CTCCCTTTGTTGTGTTGT	Hex-AGATGTCTTGTGCTGCCGGTA-BHQ-1	51
Paris	IP4	GGTAACTGGTATGATTTCG	CTGGTCAAGGTTAATATAGG	FAM-TCATACAAACCACGCCAGG-BHQ-1	
Japan	Japan-NIID-N	AAATTTTGGGGACCAGGAAC	TGGCAGCTGTGTAGGTCAAC	FAM-ATGTCGCGCATTGGCATGGA-BHQ1	52

References

27 Corman, V.M.; Landt, O.; Kaiser, M.; Molenkamp, R.; Meijer, A.; Chu, D.K.; Bleicker, T.; Brunink, S.; Schneider, J.; Schmidt, M.L.; et al. Detection of 2019 novel coronavirus (2019-nCoV) by real-time RT-PCR. *Euro. Surveill.* **2020**, *25*, 2000045. https://doi.org/10.2807/1560-7917.ES.2020.25.3.2000045.

50 Diagnostic Detection of Novel Coronavirus 2019 by Real Time RT-PCR. Available online: https://www.who.int/docs/default-source/coronaviruse/conventional-rt-pcr-followed-by-sequencing-for-detection-of-ncov-rirl-nat-inst-health-t.pdf?sfvrsn=42271c6d_4 (accessed on 21 September 2023).

51 Real-Time RT-PCR Assays for the Detection of SARS-CoV-2. Available online: https://www.who.int/docs/default-source/coronaviruse/real-time-rt-pcr-assays-for-the-detection-of-sars-cov-2-institut-pasteur-paris.pdf?sfvrsn=3662fcb6_2 (accessed on 21 September 2023).

52 Shirato, K.; Nao, N.; Katano, H.; Takayama, I.; Saito, S.; Kato, F.; Katoh, H.; Sakata, M.; Nakatsu, Y.; Mori, Y.; et al. Development of Genetic Diagnostic Methods for Detection for Novel Coronavirus 2019 (nCoV-2019) in Japan. *Jpn J. Infect. Dis.* **2020**, *73*, 304–307.

54 Chu, D.K.W.; Pan, Y.; Cheng, S.M.S.; Hui, K.P.Y.; Krishnan, P.; Liu, Y.; Ng, D.Y.M.; Wan, C.K.C.; Yang, P.; Wang, Q.; et al. Molecular Diagnosis of a Novel Coronavirus (2019-nCoV) Causing an Outbreak of Pneumonia. *Clin. Chem.* **2020,** *66*, 549–555.

55 Specific Primers and Probes for Detection 2019 Novel Coronavirus. Available online: https://ivdc.chinacdc.cn/kyjz/202001/t20200121_211337.html (accessed on 21 September 2023).

61 CDC. Coronavirus Disease 2019 (COVID-19). Centers for Disease Control and Prevention. Available online: https://www.cdc.gov/coronavirus/2019-ncov/index.html (accessed on 21 September 2023).

62 Health TMoP. Diagnostic detection of Novel Coronavirus 2019 by Real Time RT-PCR. 2020. Available online: https://www.who.int/docs/default-source/coronaviruse/protocol-v2-1.pdf (accessed on 21 September 2023).

Supplementary Table S2. Samples submitted after Jan 1, 2022, with mutations occurring in both HKU-ORF1b and HKU-N primers/probe binding sites.

sample	location	time	HKU-ORF1b	HKU-N
EPI_ISL_892024	Japan	2022-01-07	18859:G->T	29253:C->T
EPI_ISL_9210836	Japan	2022-01-04	18854:G->A	29247:C->T
EPI_ISL_9229990	France	2022-01-04	18905:G->A	29146:A->G
EPI_ISL_9437066	Japan	2022-01-16	18854:G->A	29247:C->T
EPI_ISL_9472680	USA	2022-01-18	18788:C->T	29254:G->T
EPI_ISL_9949108	Spain	2022-02-07	18905:G->A	29250:C->T
EPI_ISL_10444027	Bulgaria	2022-01-06	18788:C->A	29183:A->C
EPI_ISL_10444026	Bulgaria	2022-01-06	18788:C->A	29183:A->C
EPI_ISL_10667821	Japan	2022-01-17	18854:G->A	29247:C->T
EPI_ISL_10667820	Japan	2022-01-17	18854:G->A	29247:C->T
EPI_ISL_10667814	Japan	2022-01-15	18854:G->A	29247:C->T
EPI_ISL_10749631	Germany	2022-02-14	18795:C->T	29249:C->T
EPI_ISL_10807455	Japan	2022-01-19	18854:G->A	29247:C->T
EPI_ISL_11300409	Japan	2022-01-14	18854:G->A	29247:C->T
EPI_ISL_11372287	France	2022-01-08	18905:G->A	29253:C->T
EPI_ISL_11450704	India	2022-01-03	18906:T->C	29149:C->T
EPI_ISL_11479776	Germany	2022-03-12	18855:T->C	29149:C->T
EPI_ISL_12400105	Denmark	2022-04-05	18788:C->T	29247:C->T
EPI_ISL_12451374	Luxembourg	2022-04-11	18788:C->T	29185:T->A
EPI_ISL_12703300	Luxembourg	2022-04-23	18849:T->C	29244:T->C
EPI_ISL_13014191	Germany	2022-05-19	18855:T->C	29149:C->T
EPI_ISL_12858463	USA	2022-05-10	18788:C->T	29185:T->A
EPI_ISL_12708054	USA	2022-05-05	18788:C->T	29185:T->A
EPI_ISL_12707433	USA	2022-05-04	18788:C->T	29185:T->A
EPI_ISL_13134757	Malaysia	2022-05-17	18859:G->T	29197:C->T
EPI_ISL_13297846	Italy	2022-06-07	18788:C->T	29253:C->T
EPI_ISL_13389585	Belgium	2022-05-27	18795:C->T	29179:G->T
EPI_ISL_13463610	Panama	2022-06-04	18795:C->T	29179:G->T
EPI_ISL_14541159	Japan	2022-07-31	18888:C->T	29179:G->T
EPI_ISL_15083835	Italy	2022-09-19	18796:C->T	29247:C->T
EPI_ISL_15149196	Germany	2022-09-16	18907:G->T	29250:C->A
EPI_ISL_15867442	Russia	2022-04-23	18795:C->T	29151:G->A
EPI_ISL_16024682	Sweden	2022-11-25	18792:T->C	29250:C->T
EPI_ISL_16027399	Panama	2022-05-28	18795:C->T	29179:G->T
EPI_ISL_17004583	Germany	2023-02-09	18788:C->T	29149:C->T
EPI_ISL_17069351	Germany	2023-02-16	18788:C->T	29149:C->T
EPI_ISL_17069245	Germany	2023-02-16	18788:C->T	29149:C->T
EPI_ISL_17090748	South Korea	2023-02-05	18863:T->G	29249:C->T
EPI_ISL_17094806	United Kingdom	2023-02-19	18855:T->C	29185:T->A
EPI_ISL_17294438	South Korea	2023-03-04	18863:T->G	29239:G->A
EPI_ISL_16211697	Spain	2022-05-05	18888:C->A	29162:G->T

EPI_ISL_17473524	South Korea	2023-03-23	18891:G->A	29148:T->C
EPI_ISL_17605431	Canada	2023-04-12	18855:T->C	29185:T->A
EPI_ISL_17765614	Australia	2023-05-09	18855:T->C	29247:C->T

Supplementary Table S3. Mutation ratio for commonly used assays and double mutation ratio for dual-assays.

Assay	Forward Primer(%)	Reverse primer(%)	Probe(%)	Double mutation count/total sample
ChinaCDC-ORF1ab	0.29407	0.18063	0.32947	16434/2451477
ChinaCDC-N	85.71816	37.23029	0.29256	
Charite-RdRP	24.99228	0.17267	0.20110	59165/2451477
Charite-E	19.63616	0.07200	0.11063	
HKU-ORF1b	0.32980	0.55575	0.14171	588/2451477
HKU-N	0.46237	1.36028	1.47368	
USACDC-N1	3.26758	0.54082	20.85547	
USACDC-N2	0.96509	0.47918	1.36616	2048/2451477
USACDC-N3	1.57787	0.93021	1.51945	
Thailand-N	1.75874	18.36530	0.35456	
IP2	0.05621	1.57317	0.57635	1184/2451477
IP4	0.06555	0.74816	4.93123	
Japan-NIID-N	0.54579	1.03109	0.43990	

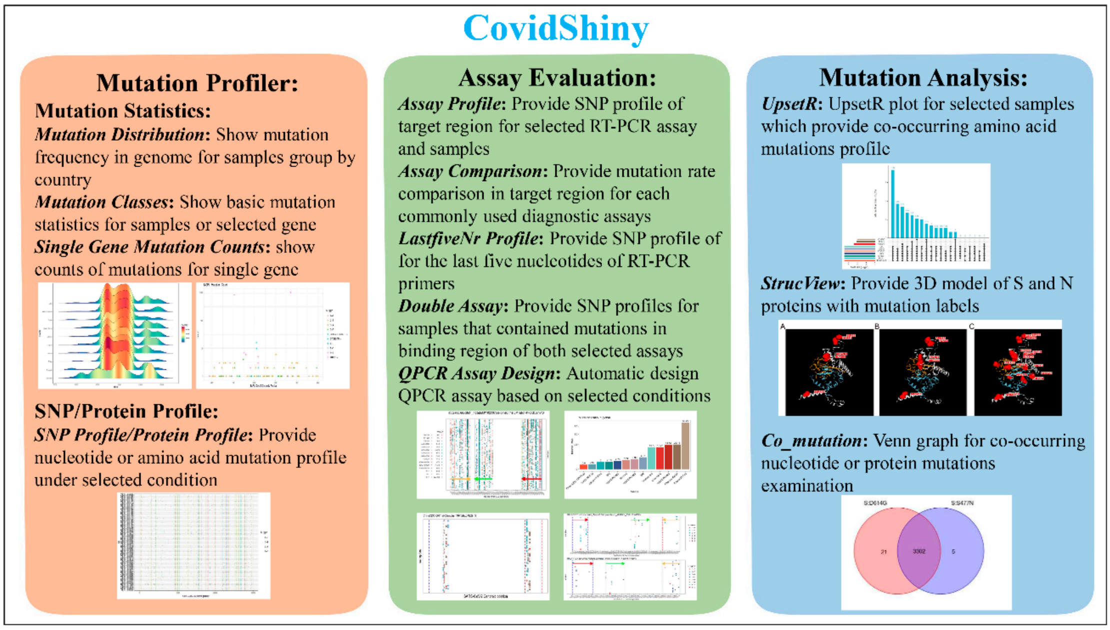

Supplementary Figure S1. Overview and brief annotation for all modules and functions of CovidShiny

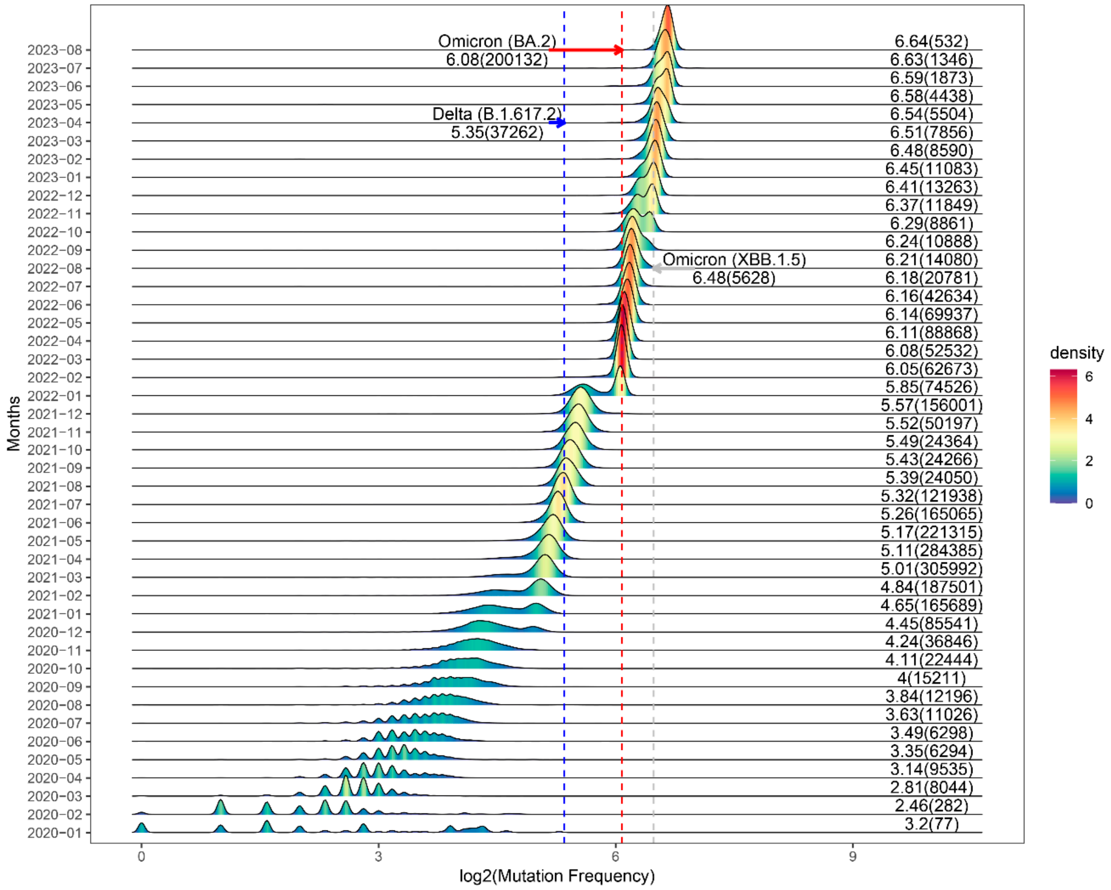

Supplementary Figure S2. The density ridgeline plot of mutation frequency accumulation since 2020-01. The number shown on the right side of the plot is the log2 transformed average mutation counts of SARS-CoV-2 in a month (with a total sample number per month in brackets). Dash lines indicate the average log2 transformed average mutation counts of three variants, the value is labeled on the site with the sample number in bracket. (Blue line: Delta(B.1.617.2), Red line: Omicron(BA.2), Grey line: Omicron(XBB.1.5))
